# Total Psoas Area and Psoas Density Assessment in COVID-19 Patients Using CT Imaging – Could Muscle Mass Alteration During Intensive Care Hospitalization be Determined?

**DOI:** 10.2478/jccm-2023-0026

**Published:** 2023-11-14

**Authors:** Oana Elena Branea, Anca Gabriela Vlad, Mihai Pui, Diana Andreea Becica, Mihai Emanuel Becica, AnaMaria Romina Budeanu, Razvan Gabriel Budeanu, Florin Stoica, Alexandra Elena Lazar

**Affiliations:** George Emil Palade University of Medicine, Pharmacy, Science, and Technology of Targu Mures, Romania; Targu Mures County Emergency Clinical Hospital, Romania

**Keywords:** intensive care, psoas muscle, CT scan, critically ill, COVID-19

## Abstract

**Background:**

Since its debut, as reported by the first published studies, COVID-19 has been linked to life-threatening conditions that needed vital assistance and admission to the intensive care unit. Skeletal muscle is a core element in an organism’s health due to its ability to keep energy balance and homeostasis. Many patients with prolonged hospitalization are characterized by a greater probability prone to critical illness myopathy or intensive care unit-acquired weakness.

**Objective:**

The main aim of this study was to assess the skeletal muscle in a COVID-19 cohort of critically ill patients by measuring the psoas area and density.

**Material and methods:**

This is a retrospective study that included critically ill adult patients, COVID-19 positive, mechanically ventilated, with an ICU stay of over 24 hours, and who had 2 CT scans eligible for psoas muscle evaluation. In these patients, correlations between different severity scores and psoas CT scans were sought, along with correlations with the outcome of the patients.

**Results:**

Twenty-two patients met the inclusion criteria. No statistically significant differences were noticed regarding the psoas analysis by two blinded radiologists. Significant correlations were found between LOS in the hospital and in ICU with psoas area and Hounsfield Units for the first CT scan performed. With reference to AUC-ROC and outcome, it is underlined that AUC-ROC is close to 0.5 values, for both the psoas area and HU, indicating that the model had no class separation capacity.

**Conclusion:**

The study suggested that over a short period, the psoas muscle area, and the psoas HU decline, for both the left and the right sight, in adult COVID-19 patients in ICU conditions, yet not statistically significant. Although more than two-thirds of the patients had a negative outcome, it was not possible to demonstrate an association between the SARS-COV2 infection and psoas muscle impairment. These findings highlight the need for further larger investigations.

## Introduction

Since its debut, as reported by the first published studies, COVID-19 has been linked to life-threatening conditions that needed vital assistance and admission to the intensive care unit (ICU) [1,2]. Although the medical picture of those diagnosed with COVID-19 appeared to be heterogeneous [3], a large group of patients experienced severe COVID-19 infection prompting life-saving treatments such as non-invasive ventilation (NIV), invasive mechanical ventilation (IMV), vasopressors, extracorporeal membrane oxygenation, and renal replacement therapy [4]. Considering acute respiratory failure (ARF) the indicator for fulminant COVID-19 disease, supportive care of hypoxic distress was the principal part of the therapy [3,4]. Therefore, skeletal muscle-related problems were situated on a secondary level. However, skeletal muscle is a core element in an organism’s health due to its ability to keep energy balance and homeostasis. About 45–50% of the body’s mass consists of skeletal muscle, which is essential for physical activity, oxygen consumption, energy metabolism, storage, and turnover [5]. For instance, patients with acute COVID-19 viral infection may suffer from mild to severe acute myopathy [6,7]. Additionally, many patients with prolonged hospitalization [8], are characterized by a greater probability prone of critical illness myopathy (CIM) or intensive care unit-acquired weakness (ICUAW) [9]. Nonetheless, skeletal muscle changes rooted in low muscle strength, low muscle quantity, and low physical performance represent the attributes of sarcopenia. Despite various techniques used to estimate muscle mass or muscle quantity, computed tomography (CT) is accepted as the gold standard for non-invasive evaluation [10]. Doctors may assess muscle area and density in various patients who have previously had CT imaging. This analysis is essential for aiding doctors in dividing patients for additional issues and mortality. Only a few researchers have investigated this issue [11].

## Materials and methods

The methods pattern for the present study design considered the guidelines for reporting observational studies, The Strengthening the Reporting of Observational Studies in Epidemiology (STROBE) Statement (Table 1).

**Table 1.** STROBE checklist for the study’s methods

Study design	Cohort, observational, retrospective and monocentric
Setting	Anaesthesia and Intensive Care Unit, Târgu Mureș Emergency County Clinical Hospital, Romania Period of recruitment: August 2020 – December 2022 Exposure: COVID-19 Follow-up: two psoas CT scans Data collection: electronic medical and imagistic records
Participants	Eligibility criteria: ≥ 18 years of age admitted in the ICU for at least 24 hours, positively tested for SARS-COV2 by real-time polymerase chain reaction (RT-PCR), and who had two eligible CT scans for psoas analysis
Variables	Main outcomes: analysis of the changes in psoas area and psoas HU in critically ill Predictors: length of stay in hospital, length of stay in ICU, mechanical ventilation Diagnostic criteria: psoas analysis by CT scan
Data sources/measurement	Data sources: Picture Archiving and Communicating System and RadiAnt DICOM Viewer Measurement: Psoas muscle area and density (HU) were measured at the level of the third lumbar vertebrae
Study size	Inclusion of critically ill patients who were hospitalized in the ICU for more than 24h, adults, that had two eligible CT scans for psoas assessment
Statistical methods	To conduct the statistical analysis, SPSS, version 27 (SPSS Inc. Chicago, IL, USA) was utilized. Continuous data are presented as medians (minimum-maximum) or means (±standard deviation) and categorical data as proportions. The Kolmogorov–Smirnov test was used to assess the normal distribution of continuous numerical variables. The Mann-Whitney U test was used for non-Gaussian variables, and Student’s t-test was used for the Gaussian continuous variables. Inter-variability is performed by Bland and Altman methods. Receiver operating characteristics curve analysis was performed. Area under the curve values were displayed including a 95% confidence interval. For correlations, Spearman’s coefficients were used. A p-value of less than 0.05 was considered statistically significant.

### Study design

This monocentric, observational, retrospective study was conducted in the Anesthesia and Intensive Care Unit of Târgu Mureș Emergency Clinical County Hospital, Romania. The data were collected for the period August 2020 – December 2022 and patients with SARS-COV2 infection were identified by systematic analysis of the ICU database. The Ethics Committee approved the study protocol (No. 2083/15.02.2023). Collected data were anonymous and informed consent was waived for all patients.

### Study participants

**Fig.1.**
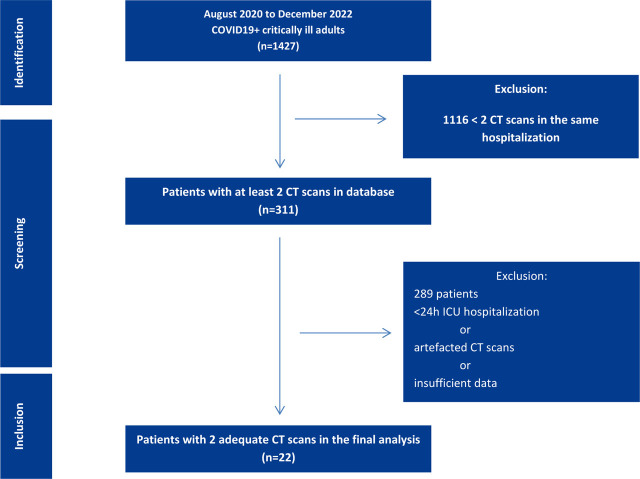
Flow chart

The screening included critically ill adult patients. Inclusion criteria were patients ≥ 18 years of age admitted in the ICU for at least 24 hours, positively tested for SARS-COV2 by real-time polymerase chain reaction (RT-PCR), and who had two eligible CT scans for psoas analysis. Non-critically ill patients, or those tested negative for SARS-COV2, minors, pregnant women, patients hospitalized for less than 24 hours in the ICU or those with non-eligible CT scans for psoas analysis were excluded. The final cohort included twenty-two patients, for whom data was collected from the electronic medical records (Hipocrate3 Concept) (Figure 1).

### Study objectives

The primary aims were to analyze and compare the psoas area and psoas Hounsfield units (HU) in two CT scans operated during the hospitalization. Clinical and biological characteristics, along with outcomes in patients with SARS-COV2 infection were evaluated. Other studied parameters included: severity scores, length of stay (LOS) in the hospital and in the ICU, methods of oxygenation (low-flow and high-flow oxygen, noninvasive ventilation (NIV), invasive mechanical ventilation (IMV), and admission laboratory results. In the hospital and in the ICU, the mortality rate was recorded.

### Severity scores assessment

Multiple instruments have been developed as triage tools using clinical and paraclinical parameters that critical care professionals may use to find high-risk COVID-19 patients. In this study, the following severity scores were explored:

– quick Sequential Organ Failure Assessment (qSOFA), as an index to calculate the risk of sepsis-related morbidity and death [12];
– quick COVID-19 Severity Index (qCSI), as an index to evaluate the risk of acute respiratory distress during the next 24 hours [13];
– COVID Home Safely Now (CHOSEN) Risk Score for COVID-19 and modified CHOSEN score, as indexes to predict if COVID-19 patients will be suitable for discharge [14].

### Psoas CT evaluation

**Fig. 2.**
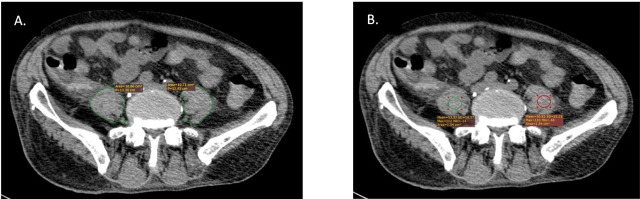
CT psoas muscle evaluation at the third lumbar vertebrae. A. Cross-sectional area evaluation showing muscle area. B. Evaluation of muscle density in Hounsfiled unit.

Two radiologists with at least two years of experience each evaluated the scans. Radiologists were unaware of the clinical progress and results of the patients. Patients with two psoas examinations were included in the study group to observe the musculoskeletal changes. The patients were lying supine and the CT images were evaluated in the abdominal window. Psoas muscle area and density (HU) were measured at the level of the third lumbar vertebrae (Figure 2). Picture Archiving and Communicating System (PACS) and RadiAnt DICOM Viewer were used for data analysis.

### Statistical analysis

Excel sheets served to arrange and statistically evaluate the data. To conduct the statistical analysis, SPSS, version 27 (SPSS Inc. Chicago, IL, USA) was utilized. Continuous data are presented as medians (minimum-maximum) or means (±standard deviation) and categorical data as proportions. The Kolmogorov–Smirnov test was used to assess the normal distribution of continuous numerical variables. The Mann-Whitney U test was used for non-gaussian variables, and Student’s t-test was used for the Gaussian continuous variables. Inter-variability is performed by Bland and Altman methods. Receiver operating characteristics (ROC) curve analysis was performed. Area under the curve (AUC) values were displayed including a 95% confidence interval (CI). For correlations, Spearman’s coefficients were used. A p-value of less than 0.05 was considered statistically significant.

## Results

Patients’ characteristics, details about admission status, inflammatory markers, and metabolic response are summarized in Table 2. Out of 22 patients included, one-third were male (36.4%), with a mean age of 71.09±10.57 years old. All patients had an emergency criterion to be admitted to the ICU and half of them were admitted for ARF. Used as a prognostic tool for early clinical decompensation, qCSI identified patients at risk of critical respiratory illness in the next 24 hours (qCSI≥7 points). At the same time, CHOSEN score (<29 points) and modified CHOSEN score (<19 points), used as predictors of suitability for discharge, identified patients not apt to be discharged. Median LOS in hospital was 18 days and median LOS in ICU was 11 days. When admitted to the ICU 50% of the patients needed IMV and during hospitalization, the percentage increased by 30%. During the ICU stay, the majority received IMV for more than 96 hours. Laboratory results identified white blood count (WBC) values of 12.307±8.4211/μL, neutrophilia 10.381±7.924/μL, and lymphopenia 0.958±0.783/μL and high values of CRP= 106.810 (5.660–353.300) mg/L. Elevated neutrophil to lymphocytes ratio (NLR)= 10.537 (3.758–66.631), platelets to lymphocytes ratio (PLR) = 382.907 (59.644–1184.2100), and SII= 2361.589 (474.000–42981.947) showed an imbalance in inflammatory status. The metabolic response identified normal values for creatinine of 1.025 (0.140–8.160) mg/dL and glucose of 149.136±73.879 mg/dL in critical care settings.

**Table 2.** Patients’ characteristics

**Demographics**	**All patients (n=22)**
Male sex (n, %)	8 (36.4%)
Age (years, mean±SD)	71.09±10.57

**ICU admission diagnostics**	

ARF (n, %)	11 (50%)
Other (n, %)	11 (50%)

**Severity scores**	

qCSI (mean±SD)	8.73±2.27
CHOSEN score (median (min-max))	13 (1–44)
modified CHOSEN score (mean±SD)	19.33±9.81

**Hospital stay characteristics**	

LOS in Hospital (days) (median (min-max))	18 (10–91)
LOS in ICU (days) (median (min-max))	11 (2–91)
Time period between CTpsoas1 and CT psos2 (days) (medain (min-max))	10 (3–19)

**ICU admission oxygenation**	

Facial mask (n, %)	9 (40.9%)
NIV-CPAP (n, %)	2 (9.1%)
IMV (n, %)	11 (50%)

**ICU stay oxygenation**	

Need for NIV-CPAP (n, %)	5 (22.7%)
Need for IMV (n, %)	18 (81.8%)
IMV <24 hours	3 (13.6%)
IMV 24–96 hours	2 (9.1%)
IMV >96 hours	13 (59.1%)

**Admission laboratory report**	

WBC (mean±SD)	12.307±8.421
Neutrophils (mean±SD)	10.381±7.924
Lymphocytes (mean±SD)	0.958±0.783
Platelets (median (min-max))	262.500 (90.000–1081.000)
NLR (median (min-max))	10.537 (3.758–66.631)
PLR (median (min-max))	382.907 (59.644–1184.2100)
SII (median (min-max))	2361.589 (474.000–42981.947)
CRP (median (min-max))	106.810 (5.660–353.300)
Creatinine (median (min-max))	1.025 (0.140–8.160)
Glucose (mean±SD)	149.136±73.879

ICU: Intensive Care Unit; ARF: acute respiratory failure; qCSI: quick COVID-19 Severity Index; CHOSEN score: COVID Home Safely Now; LOS: length of stay; NIV-CPAP: non-invasive ventilation-continuous positive airway pressure; IMV: invasive mechanical ventilation; WBC: white blood cells (normal range 3.6–10 10^3^ /μL); Neutrophils (normal range 1.4–6.5 10^3^ /μL); Lymphocytes (normal range 1.2–3.4 10^3^ / μL); Platelets (normal range 150–450 10^3^ /μL) NLR: neutrophil to lymphocyte ratio; PLR: platelet to lymphocyte ration; SII: systemic immune-inflammation index = neutrophil x platelet/lymphocyte; CRP: C reactive protein (normal range 0–5 mg/L); Creatinine (mg/dL, normal range 0.7–1.20 mg/dL).Glucose (g/dL, ≥180g/dL high serum glucose), SD: standard deviation

**Fig. 3.**
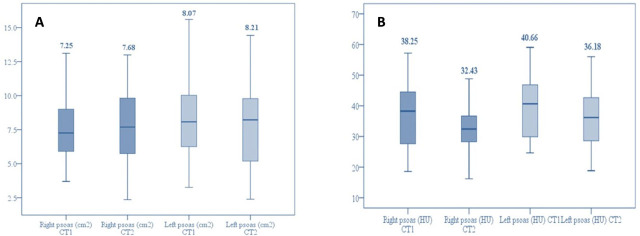
Box-plot comparing right and left changes in psoas muscle area (A) and psoas muscle HU (B).

Minor and not statistically significant differences (p> 0.05) were noticed regarding the right and the left total psoas area (TPA) and HU when comparing the results of the two radiologists (Table 3). Further analysis included the mean results of the two radiologists for each CT scan evaluation. As shown in Table 4., it is noticed that the right TPA decreased by −0.12 cm^2^ and the left TPA decreased by −0.59 cm^2^. Changes were observed in the right psoas HU by −4.24 HU and in the left psoas HU by −3.99 HU. There are no statistically significant differences, and this evidence is also sustained by the box plots comparing alterations over time (Figure 3).

**Table 3.** Assessment of total psoas area and psoas HU in the first and second CT scan

**CT scan parameter**	**Investigator A Mean±SD**	**Investigator B Mean±SD**	**Mean Difference**	**p difference A and B**
Right TPA1(cm^2^)	8.01±2.70	7.72±2.97	−0.29	0.73
Right TPA2(cm^2^)	8.39±3.19	7.08±2.92	−1.31	0.15
Left TPA1(cm^2^)	8.71±3.80	8.41±3.42	−0.29	0.78
Left TPA2(cm^2^)	8.19±3.20	7.70±3.27	−0.48	0.61
Right psoasHU1	35.95±10.86	39.34±10.27	3.38	0.28
Right psoasHU2	30.55±13.00	36.40±10.65	5.83	0.10
Left psoasHU1	38.49±10.35	41.86±10.99	3.37	0.29
Left poasHU2	34.68±11.99	37.86±9.69	3.18	0.33

CT: computed tomography; TPA1: total psoas area in the first CT scan; TPA2: total psoas area in the second CT scan; HU: Hounsfield units in the first CT scan; HU2: Hounsfield units in the second CT scan; SD: standard deviation

**Table 4.** Dynamic changes in total psoas area and psoas HU in two CT scan evaluations

**CT scan parameter**	**CT1 (A+B) Mean±SD**	**CT2 (A+B) Mean±SD**	**Difference Mean**	**p difference 1^st^ and 2^nd^**
Right TPA(cm^2^)	7.82±2.76	7.70±2.90	−0.12	0.88
Left TPA(cm^2^)	8.52±3.56	7.92±3.20	−0.59	0.56
Right psoas HU	37.4±10.21	33.16±10.31	−4.24	0.17
Left psoas HU	39.9±10.06	35.93±9.33	−3.99	0.17

CT1: mean results of investigator A and investigator B for the first CT scan; CT2: mean results of investigator A and investigator B for the second CT scan; TPA: total psoas area; HU: Hounsfield units; SD: standard deviation

**Fig. 4.**
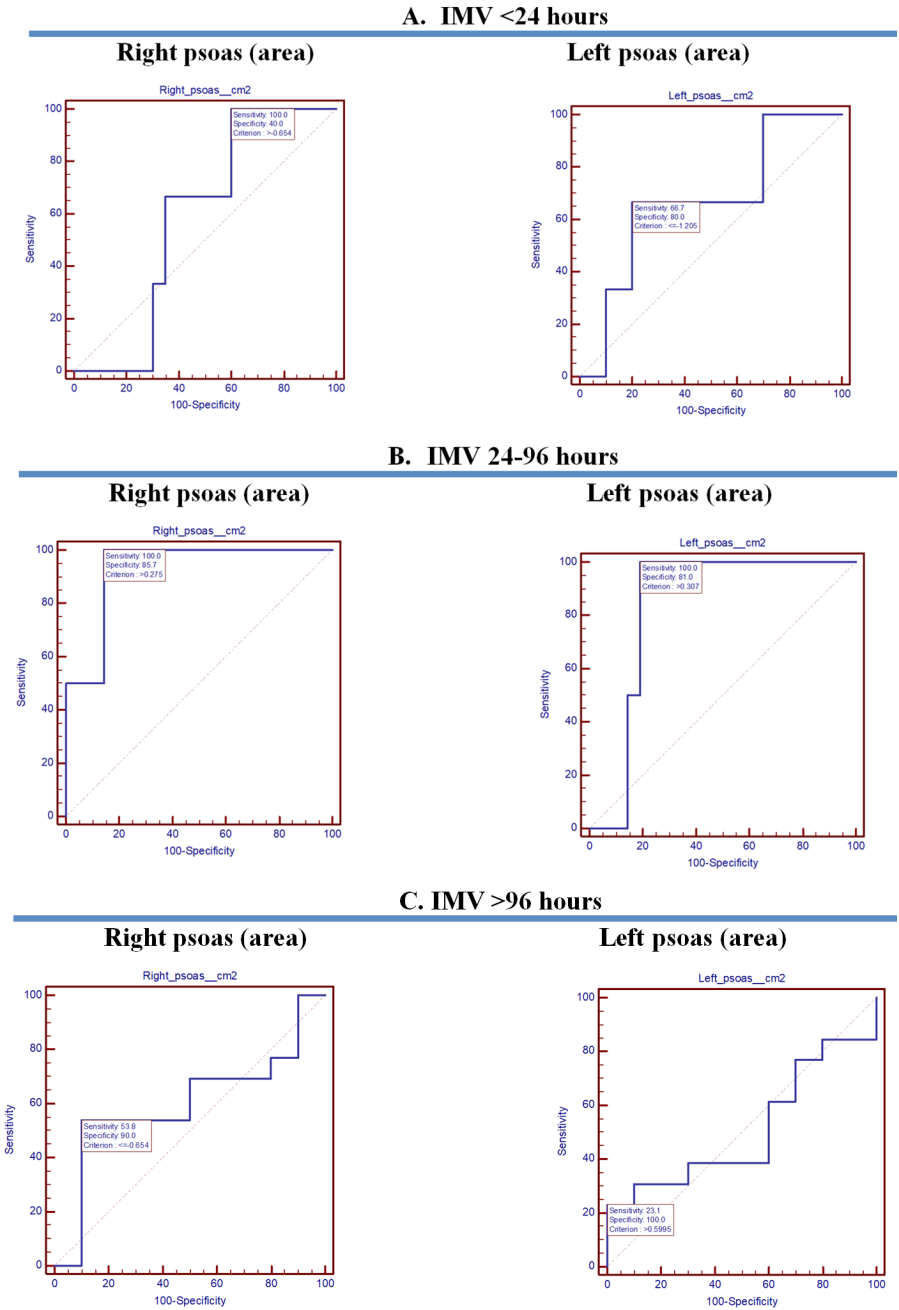
AUC-ROC for psoas area and IMV. A. IMV <24 hours. B. IMV 24-96 hours. C. IMC >96 hours.

**Fig. 5.**
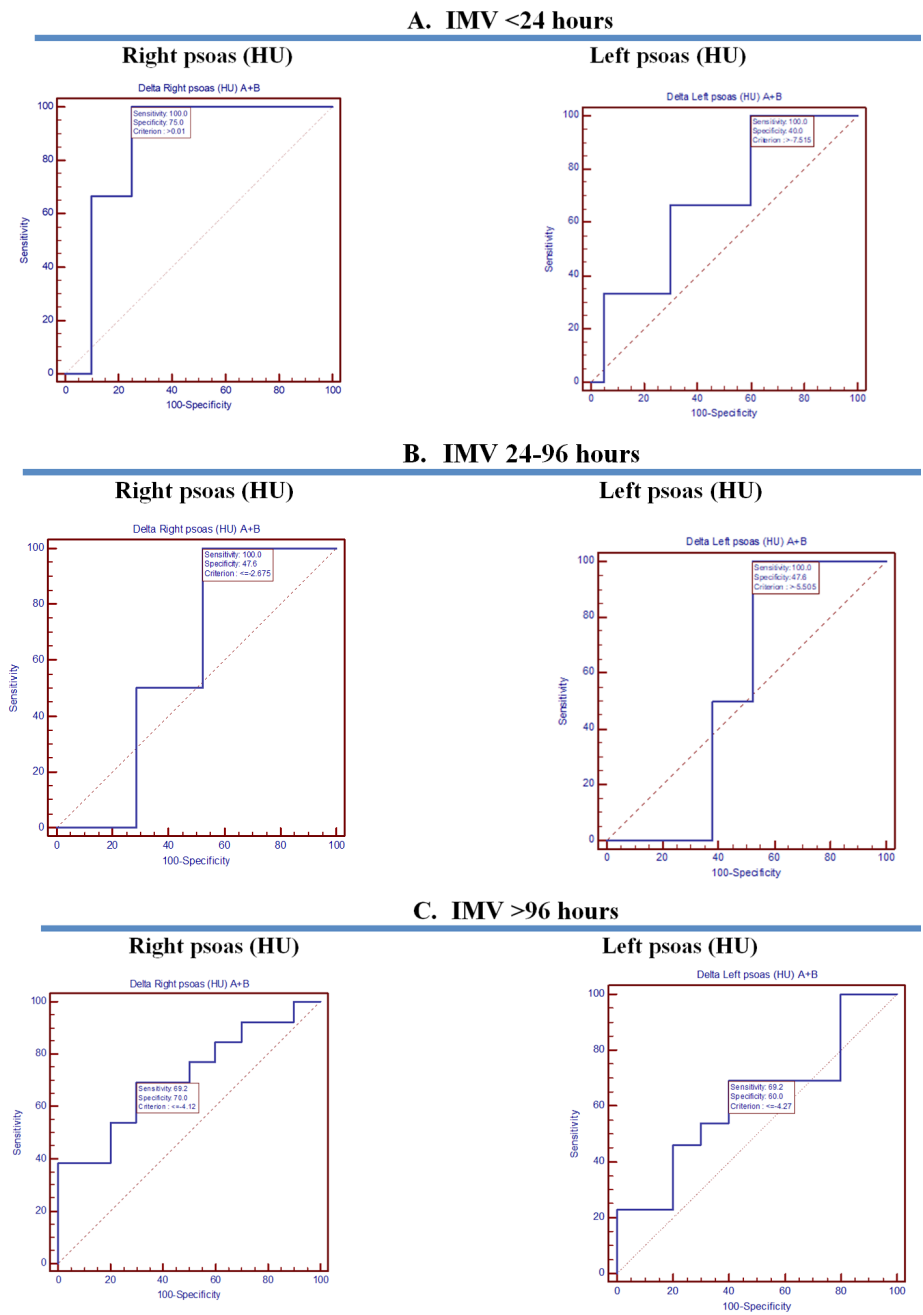
AUC-ROC for psoas HU and IMV. A. IMV <24 hours. B. IMV 24-96 hours. C. IMC >96 hours.

**Fig. 6.**
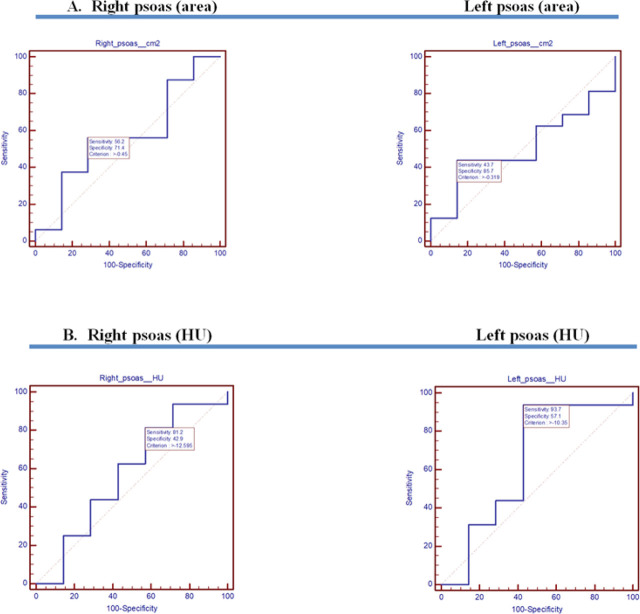
Right and left psoas analysis (area - A and HU - B) linked to the patients outcome

Along with these results, AUC-ROC for psoas area and IMV duration (Figure 4) and outcome (Figure 6), respectively AUC for psoas HU and IMV duration (Figure 5) and outcome (Figure 6) were placed side by side. Regarding AUC-ROC for psoas area and IMV duration, an AUC-ROC near 1 was obtained for right psoas area (AUC-ROC= 0.929, 95% CI= 0.741–0.994, p= 0.0001) and left psoas area (AUC-ROC= 0.833, 95% CI= 0.621–0.954, p=0.0001) and IMV duration of 24–96 hours (Table 5). As to AUC-ROC for psoas HU and >96 hours of IMV, the results were AUC-ROC= 0.715, 95% CI= 0.491–0.882, p= 0.049 for the right psoas muscle and AUC-ROC= 0.623, 95% CI= 0.399–0.814, p= 0.31 for the left psoas muscle (Table 6). With reference to AUC-ROC and outcome, it is underlined that AUCROC is close to 0.5 value, for both the psoas area and HU, indicating that the model had no class separation capacity (Table 7).

**Table 5.** AUC-ROC for the Psoas area and IMV duration

**IMV duration (hours)**	**Psoas area (cm^2^)**	**Criterion**	**AUC-ROC**	**95% CI**	**p**	**Sn**	**95% CI**	**Sp**	**95% CI**
<24	Right	>−0.654	0.583	0.362–0.783	0.53	100.00	29.2–100.0	40.00	19.1–63.9
	Left	≤−1.205	0.667	0.442–0.847	0.39	66.67	9.4–99.2	80.00	56.3–94.3

24–96	Right	>0.275	0.929	0.741–0.994	0.0001	100.00	15.8–100.0	85.71	63.7–97.0
	Left	>0.307	0.833	0.621–0.954	0.0001	100.00	15.8–100.0	80.95	58.1–94.6

>96	Right	≤−0.654	0.600	0.377–0.796	0.43	53.85	25.1–80.8	90.00	55.5–99.7
	Left	>0.599	0.508	0.294–0.720	0.951	23.08	5.0–53.8	100.00	69.2–100.0

AUC-ROC: Area Under the Curve Receiver Operating Characteristics: Sn: Sensitivity; Sp: Specificity; CI: Confidence Interval; IMV: invasive mechanical ventilation

**Table 6.** AUC-ROC for psoas HU and IMV duration

**IMV duration (hours)**	**Psoas (HU)**	**Criterion**	**AUC-ROC**	**95% CI**	**P**	**Sn**	**95% CI**	**Sp**	**95% CI**
<24	Right	>−0.01	0.850	0.640–0.963	0.0001	100.00	29.2–100.0	75.00	50.9–91.3
	Left	>−7.515	0.683	0.458–0.859	0.29	100.00	29.2–100.0	40.00	19.1–63.9

24–96	Right	≤−2.675	0.595	0.373–0.792	0.53	100.00	15.8–100.0	47.62	25.7–70.2
	Left	>−5.505	0.548	0.329–0.754	0.70	100.00	15.8–100.0	47.62	25.7–70.2

>96	Right	≤−4.12	0.715	0.491–0.882	0.049	69.23	38.6–90.9	70.00	34.8–93.3
	Left	≤−4.27	0.623	0.399–0.814	0.31	69.23	38.6–90.9	60.00	26.2–87.8

AUC-ROC: Area Under the Curve Receiver Operating Characteristics: Sn: Sensitivity; Sp: Specificity; CI: Confidence Interval; IMV: invasive mechanical ventilation; HU: Hounsfield units

**Table 7.** AUC-ROC for the negative outcome (deceased patients)

**CT scan parameter**	**Criterion**	**AUC-ROC**	**95% CI**	**P**	**Sn**	**95% CI**	**Sp**	**95% CI**
Right psoas area	>−0.45	0.571	0.351–0.773	0.610	56.2	29.9–80.2	71.4	29.0–96.3
Right psoas HU	>−12.59	0.571	0.350–0.773	0.633	81.2	54.4–96.0	42.9	9.9–81.6
Left psoas area	>−0.319	0.509	0.295–0.721	0.944	43.7	19.8–70.1	85.7	42.1–99.6
Left psoas HU	>−10.35	0.643	0.418–0.829	0.362	93.7	69.8–99.8	57.1	18.4–90.1

CT: computed tomography; HU: Hounsfield units; AUC-ROC: Area Under the Curve Receiver Operating Characteristics: Sn: Sensitivity; Sp: Specificity; CI: Confidence Interval

Assessing possible correlations between LOS in hospital and in ICU with psoas area and HU, for the first CT scan performed, it is shown a positive coefficient correlation (0.417) and statistically significant (p= 0.02) with regard to the LOS in ICU and right psoas HU, and a positive coefficient correlation (0.666) and statistically significant (p= 0.01) with regard to the LOS in ICU and left psoas HU (Table 8). Other than those findings, no other correlations were verified.

**Table 8.** LOS in hospital and LOS ICU correlations with psoas area and HU

**CT scan (mean A+B)**	**LOS**	**Right psoas (cm^2^)**		**Right psoas (HU)**		**Left psoas (cm^2^)**		**Left psoas (HU)**	
		**Correlation coefficient**	**P**	**Correlation coefficient**	**p**	**Correlation coefficient**	**p**	**Correlation coefficient**	**p**
CT scan1	Hospital	0.015	0.94	0.202	0.36	−0.054	0.810	0.362	0.09
	ICU	−0.349	0.11	0.417	0.02	−0.322	0.11	0.666	0.01

CT scan2	Hospital	−0.087	0.70	−0.198	0.37	−0.069	0.76	−0.007	0.97
	ICU	−0.307	0.16	0.007	0.97	−0.263	0.23	0.250	0.26

CT: computed tomography; LOS: length of stay; ICU: intensive care unit; HU: Hounsfield units

## Discussions

In the long run, it was found that 48% of patients may gain ICU-AW ^[15]^. Critically ill patients may lose more than 15% of their muscle mass over one week and 2% per day in the first week of admission, which could lead to negative long-term repercussions, as underlined in one of the most recent systematic reviews and meta-analyses by Fazzini et al. [15,16]. Typically, ICU-AW is assessed by ultrasound and CT [15].

In our study, we analyzed two CT scans performed during the hospitalization and we compared TPA and psoas HU over a period (Figure 3). Firstly, good reliability in both psoas measurements was demonstrated by the two blinded radiologists. With minor mean differences in right and left TPA and psoas HU, no statistically significant differences were noticed (p>0.05) (Table 3.) Secondly, the dynamic decrease was detected in TPA (right TPA= −0.12cm^2^, p= 0.88; left TPA= −0.59cm^2^, p= 0.56) and in psoas HU (right psoas HU= −4.24, p=0.17; left psoas HU= −3.99, p= 0.17), but not statistically significant as exposed in Table 4.

Furthermore, AUC-ROC for psoas area and IMV was calculated, classifying patients <24 hours of IMV, 24–96 hours of IMV, and > 96 hours of IMV. As shown in Table 5, a statistically significant result was found for patients ventilated for a maximum of 96 hours, as follows for the right psoas area was obtained: criterion >0.275, AUC-ROC= 0.929, 95%CI= 0.741–0.994, p= 0.0001 and for the left psoas area: was obtained criterion >0.307, AUC-ROC= 0.833, 95%CI= 0.621–0.954, p= 0.0001. As both the right and left psoas AUC-ROC is near one, it suggests a good reserve regarding this period of IMV (Figure 4).

Boutin et al. has analyzed sarcopenia imaging implications. As a follow, it has been shown that there is a strong association between CT-derived values from a single CT slice and whole-body adipose tissue and skeletal muscle [17]. Of great significance is that a CT scan can be used to assess the presence of intramuscular fat: “A threshold range of 29 to 150 HU is commonly used to define the muscle, whereas adipose tissue typically ranges from − 30 to − 190 Hounsfield units” ^[17,18]^. Even if there is no statistically significant evidence, our study results identified for both A and B investigators that right psoas HU and left psoas HU are in the range that defines the muscle (Table 3.). Assessing the same intervals of IMV as for the TPA, regarding psoas HU, the following results were found for >96 h of IMV: for the right psoas HU, criterion ≤−4.12, AUC-ROC= 0.715, 95%CI= 0.491–0.882, p= 0.049; for the left psoas HU, criterion ≤−4.27, AUC-ROC= 0.623, 95%CI=0.399–0.814, p= 0.31. In this situation, as AUC-ROC is close to 0.5, the model had a dissatisfactory separation capacity for IMV >96 hours (Figure 5)

Regarding the clinical presentation of COVID-19, it is complemented by multiple extrapulmonary manifestations, despite the disease’s notable respiratory complications. Subjects suffering from moderate to severe SARS-CoV-2 infection acknowledged signs of muscular impairment, reduced capacity for physical activity, and fatigue [19]. Moreover, among SARS-CoV-2 critically ill patients, acute sarcopenia was identified as a side effect [20]. We analyzed correlations between psoas changes and LOS in hospital and in ICU. Positive coefficient correlation was found for left psoas on the first CT scan (correlation coefficient= 0.362, p= 0.09 for the LOS in Hospital; correlation coefficient= 0.666, p= 0.01 for the LOS in ICU). Nonetheless, AUC-ROC for negative outcome was evaluated (Figure 6). Only six patients were discharged from the hospital. The curves show poor separation capacity for the negative outcome (death), for both TPA and psoas HU (AUC-ROC close to 0.5).

### Strengths and limitations

When weighing the variables that could affect the onset of muscle mass decrease, literature proved that infection state, treatment of severe inflammation caused on by the SARS-CoV-2 infection, prolonged immobility, and caloric intake, could all alter the expected outcome of the illness ^[20]^. In this regard, our study has some strengths and some limitations. On one hand, dynamic psoas CT scans were screened to determine TPA and psoas HU, as well as many recorded variables were analyzed. Also, two blinded radiologists participated in the analysis of the same psoas CT images, with no statistically significant differences between their results. On the other hand, our study includes a small sample size, in addition to its retrospective design. Therefore, it is not possible to demonstrate a causal relationship between the viral infection and muscle changes, respectively patients’ outcomes. Due to the lack of generalizability, requirement for more detailed insight would be necessary.

## Conclusions

In conclusion, our study suggested that over a short period the psoas muscle area and the psoas HU decline for both the left and the right sight in adult COVID-19 patients in ICU conditions. It was not noticed a statistically significant difference regarding the results of TPA and HU comparing the two CT scans in dynamics. Although more than two-thirds of the patients had a negative outcome, it was not possible to demonstrate an association between the SARS-COV2 infection and psoas muscle impairment. These findings highlight the need for further larger investigations.
